# The UCSC Genome Browser database: 2014 update

**DOI:** 10.1093/nar/gkt1168

**Published:** 2013-11-21

**Authors:** Donna Karolchik, Galt P. Barber, Jonathan Casper, Hiram Clawson, Melissa S. Cline, Mark Diekhans, Timothy R. Dreszer, Pauline A. Fujita, Luvina Guruvadoo, Maximilian Haeussler, Rachel A. Harte, Steve Heitner, Angie S. Hinrichs, Katrina Learned, Brian T. Lee, Chin H. Li, Brian J. Raney, Brooke Rhead, Kate R. Rosenbloom, Cricket A. Sloan, Matthew L. Speir, Ann S. Zweig, David Haussler, Robert M. Kuhn, W. James Kent

**Affiliations:** ^1^Center for Biomolecular Science and Engineering, School of Engineering, University of California Santa Cruz (UCSC), 1156 High Street, Santa Cruz, CA 95064, USA, ^2^Computational Biology Graduate Group, University of California Berkeley, Berkeley, CA 94720, USA, ^3^Department of Genetics, Stanford University School of Medicine, 3165 Porter Drive, Stanford, CA 94305, USA and ^4^Howard Hughes Medical Institute, Center for Biomolecular Science and Engineering, UCSC, 1156 High Street, Santa Cruz, CA 95064, USA

## Abstract

The University of California Santa Cruz (UCSC) Genome Browser (http://genome.ucsc.edu) offers online public access to a growing database of genomic sequence and annotations for a large collection of organisms, primarily vertebrates, with an emphasis on the human and mouse genomes. The Browser’s web-based tools provide an integrated environment for visualizing, comparing, analysing and sharing both publicly available and user-generated genomic data sets. As of September 2013, the database contained genomic sequence and a basic set of annotation ‘tracks’ for ∼90 organisms. Significant new annotations include a 60-species multiple alignment conservation track on the mouse, updated UCSC Genes tracks for human and mouse, and several new sets of variation and ENCODE data. New software tools include a Variant Annotation Integrator that returns predicted functional effects of a set of variants uploaded as a custom track, an extension to UCSC Genes that displays haplotype alleles for protein-coding genes and an expansion of data hubs that includes the capability to display remotely hosted user-provided assembly sequence in addition to annotation data. To improve European access, we have added a Genome Browser mirror (http://genome-euro.ucsc.edu) hosted at Bielefeld University in Germany.

## INTRODUCTION

The University of California Santa Cruz (UCSC) Genome Browser (1,2) at http://genome.ucsc.edu is a web-based resource for the scientific, medical and academic research communities that provides timely, convenient access to a database of high-quality genome sequence and annotations. The Browser tools facilitate the visualization, comparison and analysis of both hosted and user-generated data sets ranging from a genome-wide perspective down to the base level.

The Genome Browser database contains genome sequence from GenBank (3) for a wide selection of organisms, many with multiple assembly versions. In September 2013 our database included 13 primates, 33 additional mammals, 17 non-mammalian vertebrates, 13 insects, 6 worms and 5 other invertebrates. Annotation data for each genome assembly are displayed graphically as ‘tracks’ aligned to the genomic sequence and grouped according to shared characteristics, such as gene predictions or comparative genomics. The level of annotation varies among organisms. At a minimum, most assemblies offer mapping and sequence annotation tracks describing assembly, gap and GC content, and alignments of mRNA, EST and RefSeq (3) genes (available on approximately one-half of the assemblies) from GenBank. Some assemblies provide additional gene annotation tracks, such as Ensembl Genes (4) and Human Proteins, as well as multiple sequence alignments (multiz) (5) and pairwise genomic alignments between assemblies to facilitate comparative and evolutionary investigations. The heavily annotated human genome offers extensive conservation and evolutionary comparisons, a large collection of gene models including the locally generated UCSC Genes track (6,7), regulation, expression, epigenetics and tissue differentiation, variation, phenotype and disease association data, and data that have been text-mined from publications. Much of our annotation data is obtained through external collaboration. When available, links are provided to the complementary annotations in the Ensembl and NCBI browsers, and to supplementary information on other websites.

The Genome Browser serves as the repository for human and mouse genome data that was contributed through September 2012 by the Encyclopedia of DNA Elements (ENCODE) Consortium (8,9). During the transition of the ENCODE Data Coordination Center role to a joint collaboration with Stanford University, the Genome Browser team has continued to add significant new content to the ENCODE data portal (http://encodeproject.org) and publish newly reprocessed ENCODE data sets (10).

In addition to the native data sets local to the UCSC servers, the Genome Browser offers several options to users for viewing their own sequence and annotations: track and assembly data hubs, custom tracks and sessions. Alternatively, the Genome Browser database and tools may be installed on a local server for customized use (see http://genome.ucsc.edu/license/ for more information). Instructions for downloading the data, software and source code may be found at http://hgdownload.soe.ucsc.edu/downloads.html.

The following sections highlight the genome assembly and annotation data sets added to the Genome Browser since the last update in this journal and describe the significant new features and capabilities of our data access tools.

## GENOME BROWSER DATA SETS

### New genome assemblies

During the past year the UCSC team added 35 vertebrate assemblies to the Genome Browser (Table 1), including the premier releases of 20 species. In line with our focus on primates and other vertebrates, the group of newly introduced species features 4 primates (baboon, mouse lemur, squirrel monkey and tarsier), 12 additional mammals (alpaca, dolphin, ferret, hedgehog, kangaroo rat, manatee, megabat, rock hyrax, shrew, sloth, southern white rhinoceros and tree shrew) and 4 additional vertebrates (American alligator, Atlantic cod, budgerigar and coelacanth). Several of the new assemblies were added to support the generation of the 60-species Conservation track released in 2012 on the GRCm38/mm10 mouse assembly, and many of these were originally sequenced and assembled for the Mammalian Genome Project (11). We plan to release a preliminary Browser with a minimal annotation set on the new GRCh38/hg38 human assembly in late 2013 or early 2014. Beginning with this new release, the numeric portion of the UCSC human assembly version name will match the Genome Reference Consortium version number to reduce confusion.

**Table 1.** New and updated genome assemblies added to the Genome Browser since September 2012

Common name	Scientific name	Sequencing center	UCSC ID	Seq. ctr ID
Primates				
Baboon	*Papio hamadryas*	Baylor College of Medicine HGSC	papHam1	Pham_1.0
Baboon	*Papio anubis*	Baylor College of Medicine HGSC	papAnu2	Panu_2.0
Bushbaby	*Otolemur garnettii*	Broad Institute	otoGar3	OtoGar3
Chimpanzee	*Pan troglodytes*	Chimpanzee Sequencing and Analysis Consortium	panTro4	Build 2.1.4
Gibbon	*Nomascus leucogenys*	Gibbon Genome Sequencing Consortium	nomLeu2	Nleu1.1
			nomLeu3	Nleu3.0
Mouse lemur	*Microcebus murinus*	Broad Institute	micMur1	MicMur1.0
Rhesus macaque	*Macaca mulatta*	Beijing Genomics Institute	rheMac3	CR_1.0
Squirrel monkey	*Saimiri boliviensis*	Broad Institute	saiBol1	SaiBol1.0
Tarsier	*Tarsius syrichta*	Broad Institute	tarSyr1	Tarsyr1.0
Other mammals				
Alpaca	*Vicugna pacos*	Broad Institute	vicPac1	VicPac1.0
			vicPac2	VicPac2.0
Armadillo	*Dasypus novemcinctus*	Baylor College of Medicine HGSC	dasNov3	DasNov3
Cat	*Felis catus*	International Cat Genome Sequencing Consortium	felCat5	Felis_catus-6.2
Dolphin	*Tursiops truncatus*	Baylor College of Medicine HGSC	turTru2	Ttru_1.4
Ferret	*Mustela putorius furo*	Ferret Genome Sequencing Consortium	musFur1	MusPutFur1.0
Hedgehog	*Erinaceus europaeus*	Broad Institute	eriEur1	Draft_v1
Kangaroo rat	*Dipodomys ordii*	Baylor College of Medicine HGSC, Broad Institute	dipOrd1	DipOrd1.0
Manatee	*Trichechus manatus latirostris*	Broad Institute	triMan1	TriManLat1.0
Megabat	*Pteropus vampyrus*	Broad Institute	pteVam1	PteVap1.0
Naked mole rat	*Heterocephalus glaber*	Broad Institute	hetGla2	HetGla_female_1.0
Pig	*Sus scrofa*	Swine Genome Sequencing Consortium	susScr3	Sscrofa10.2
Pika	*Ochotona princeps*	Broad Institute	ochPri2	OchPri2
Rock hyrax	*Procavia capensis*	Baylor College of Medicine HGSC	proCap1	Procap1.0
Shrew	*Sorex araneus*	Broad Institute	sorAra1	SorAra1.0
Sloth	*Choloepus hoffmanni*	Broad Institute	choHof1	ChoHof1.0
Southern white rhinoceros	*Ceratotherium simum simum*	Broad Institute	cerSim1	cerSimSim1.0
Squirrel	*Spermophilus tridecemlineatus*	Broad Institute	speTri2	SpeTri2.0
Tree shrew	*Tupaia belangeri*	Broad Institute	tupBel1	Tupbel1.0
Other vertebrates				
American alligator	*Alligator mississippiensis*	Int’l Crocodilian Genomes Working Group	allMis1	allMis0.2
Atlantic cod	*Gadus morhua*	Genofisk	gadMor1	GadMor_May2010
Budgerigar	*Melopsittacus undulatus*	Genome Institute at Wash. Univ. St. Louis	melUnd1	v6.3
Coelacanth	*Latimeria chalumnae*	Broad Institute	latCha1	LatCha1
Lamprey	*Petromyzon marinus*	Genome Institute at Wash. Univ. St. Louis	petMar2	WUGSC 7.0
Nile tilapia	*Oreochromis niloticus*	Broad Institute	oreNil2	OreNil1.1

The ‘UCSC ID’ column shows the Genome Browser database designation for the genome assembly.

As the number of vertebrate assemblies deposited into GenBank increases, we continue to explore options for providing timely, maximum coverage of genome assemblies in the Genome Browser. Assembly data hubs (described below) offer a potential solution for streamlining our process for hosting genome assemblies, as well as providing our users with an easy way to visualize and share their own genome sequences in the Genome Browser.

### New and updated annotations

We added many new annotation data sets to the Genome Browser in the past year, and several existing data sets underwent major revisions. Our human and mouse assemblies, which receive the bulk of attention from our user community, are the most richly annotated. This section highlights some of the new annotation tracks released this year. See Table 2 for a complete list of recent releases.

**Table 2.** New and updated annotation data sets added to the Genome Browser between September 2012 and September 2013

Annotation track	Assembly
Human genome	
1000 Genomes Phase 1 Integrated Variant Calls	hg19
1000 Genomes Phase 1 Paired-end Accessible Regions	hg19
Affymetrix CytoScan HD Array	hg19
Coriell Cell Line Copy Number Variants	hg19
Denisova: Modern Human Derived, Sequence Reads, Variant Calls, Variant Calls from 11 Modern Human Genome Sequences	hg19
DGV: Structural Variation	hg18-19
DNaseI Hypersensitivity Uniform Peaks— ENCODE/Analysis	hg19
ENCODE Regulation: DNaseI HS Clusters, Transaction Factor ChiP-seq Clusters	hg19
GENCODE Genes v14, v17	hg19
GeneReviews	hg18-19
GRCh37 Patch 10	hg19
GWAS Catalog of Published Genome-Wide Association Studies	hg18-19
Human Gene Mutation Database (HGMD)	hg19
Leiden Open Variation Database (LOVD)	hg19
Pfam domains in UCSC Genes	hg19
Proteogenomics and GENCODE Mapping—ENCODE	hg19
qPCR Primers	hg19
Reactome v41	hg17-19
Retroposed Genes	hg19
SNPs (Build 137): All SNPs, Common SNPs, Flagged SNPs, Mult. SNPs	hg19
SNPs (Build 138): All SNPs, Common SNPs, Flagged SNPs, Mult. SNPs	hg19
Transcription Factor ChIP-seq Uniform Peaks— ENCODE/Analysis	hg19
UCSC Genes	hg19
UniProt Mutations	hg19
Mouse genome	
60-species Conservation	mm10
GRC Incident Database	mm10
GRCm38 Patch Release 1	mm10
Mouse strain variants	mm10
qPCR Primers	mm10
Reactome v.41	mm8-9
SNPs (Build 137)	mm10
UCSC Genes	mm10
Cow genome	
NumtS Nuclear Mitochondrial Sequences	bosTau6
Pig genome	
NumtS Nuclear Mitochondrial Sequences	susScr2
Multiple genomes	
Ensembl Genes	Many
Human proteins	Many
Publications track	Many

#### Gene annotations

The UCSC Genes track, which includes protein-coding genes and non-coding RNA genes from RefSeq, GenBank, CCDS (12), Rfam (13) and the tRNA Genes track (14), was updated on both the GRCh37/hg19 human and GRCm38/mm10 mouse assemblies. The human UCSC Genes set increased by 2038 transcripts to a total of 82 960 transcripts, 92% of which did not change between versions. The number of genes, defined as clusters of transcripts with overlapping exons on the same strand, increased by 621 genes to 31 848. In the mouse UCSC Genes set, the number of transcripts grew by 3702 transcripts to 59 121, with 88% remaining the same between versions. The number of genes increased by 2566 genes to 31 227. For more information on the latest methods used to generate the UCSC Genes data, refer to the description pages that accompany the tracks. We also updated the GENCODE Genes (15) track on the latest human assembly to version 17.

#### Variation data

We update our SNP annotations for the human and mouse (and occasionally for other species) whenever a new version is released by dbSNP. The latest human and mouse assemblies were updated to dbSNP Build 137 in 2012–13, and the human assembly SNP tracks were updated to dbSNP Build 138 in October 2013. The annotation includes an ‘All SNPs’ track that contains all mappings of reference SNPs to the human assembly, as well as three SNP subsets: Common SNPs (those with at least 1% minor allele frequency), Flagged SNPs (annotated by dbSNP as ‘clinical’) and Mult. SNPs (those that map to multiple genomic loci, and therefore should be viewed with suspicion). The updated tracks contain additional annotation data not included in previous dbSNP tracks, and offer coloring and filtering options for configuring the Genome Browser display.

This year we released three new tracks that describe human disease-associated genetic variation based on curated public data in the Leiden Open Variation Database (LOVD) (16), the Human Gene Mutation Database (HGMD) (17) and amino acid mutations in the UniProt database (18). We also added two annotation sets based on Phase 1 sequencing data from the 1000 Genomes Project (19). The integrated variant calls track, 1000G Ph1 Vars, shows single nucleotide variants (SNVs), indels and structural variants (SVs) that have been phased into independent haplotypes, which the Genome Browser clusters by local similarity for display. The paired-end accessible regions track, 1000G Ph1 Accsbl, shows which genome regions are more or less accessible to next-generation sequencing methods that use short, paired-end reads.

#### Comparative alignments

The 60-species multiple alignment and conservation track released in 2012 for the GRCm38/mm10 mouse assembly was the largest comparative alignment track generated by UCSC to date. In 2013, we undertook an ambitious project to produce a 100-species conservation track on the GRCh37/hg19 human assembly, released to the public in Nov. 2013. As part of this undertaking we have been evaluating software alternatives, such as Cactus (20), to extend the scalability of our multiple alignment pipeline, which has been challenged by the increasing number of species.

#### ENCODE data

In the past year UCSC has focused on improving the accessibility and usability of the ENCODE data hosted in the Genome Browser. The ENCODE Analysis Working Group (AWG) reprocessed the transcription factor ChIP-seq and DNaseI HS peak call data sets released through March 2012 using the uniform processing pipeline developed for the ENCODE Integrative Analysis effort. This reprocessing factored out many of the cross-lab differences, allowing the different data sets to be used more effectively in the same analyses. These reprocessed data sets were released on the Genome Browser as the Transcription Factor ChIP-seq Uniform Peaks track (within the ENC TF Binding super-track) and the DNaseI Hypersensitivity Uniform Peaks track (within the ENC DNase/FAIRE super-track). The new data sets that met a specific integrated quality metric defined by the AWG (http://genome.ucsc.edu/ENCODE/qualityMetrics.html) were then used to update the individual Transcription Factor ChIP-seq Clusters and Digital DNaseI Hypersensitivity Clusters tracks within the ENCODE Integrated Regulation super-track set, providing summary clustered views. ENCODE data hosted in the Browser has now been fully accessioned through the Gene Expressions Omnibus (GEO) repository (21) and cross-linked back to UCSC. We also added the ENCODE Integrative Analysis Data Hub to the Genome Browser public hubs page (http://genome.ucsc.edu/cgi-bin/hgHubConnect) to provide easy, integrated access to AWG data. Together with the Roadmap Epigenomic data track hub (22), the ENCODE data provide a comprehensive look at DNA landmarks across a large number of tissues.

The Genome Browser currently hosts a large amount of ChIP-seq data on transcription factors, many of which bind to specific DNA motifs. In late 2013, we plan to release an extension to this data type that displays the location of motifs within the peak and shows the sequence logo and matching score on the track details page for the peak.

#### Publications data

In 2012, we introduced a Publications track that shows mapped DNA and protein sequences, SNPs, cytogenetic bands and gene symbols that have been text-mined from biomedical articles in Elsevier, PubMed Central and other databases (23). In the past year we have doubled the number of research articles to more than 5 million, and now classify them into different categories (disease related, protein structure, cis-regulatory, etc.) depending on their keyword content. The categories are differentiated by color in the display, which can be filtered by categories or publishers.

#### Denisova data

In February 2013, we released a set of Denisova annotations tracks in conjunction with the publication of a paper by Meyer *et al.* (24). The sequence data were derived by applying a novel single-stranded DNA library preparation method to DNA previously extracted from 40 mg of a phalanx bone excavated from Denisova Cave in the Altai Mountains of southern Siberia. The Genome Browser tracks show mappings to the human reference sequence of high-coverage Denisova sequence reads, variant calls from sequence reads of 11 modern individuals and an archaic Denisovan individual, and mutations in the modern human lineage that rose to fixation or near fixation since the split from the last common ancestor with Denisovans, along with predicted functional effects from the Ensembl Variant Effect Predictor (25).

## GENOME BROWSER SOFTWARE UPDATES

### Track and assembly data hubs

In 2011 we introduced track data hubs (26), a means for users to import collections of their own locally hosted genome annotations into the Genome Browser where they may be organized, configured and viewed alongside native tracks. Track hubs now support four compressed binary indexed file formats: BigBed and BigWig (27), both developed at UCSC, BAM (28) and VCF/tabix (29). As genome sequencing becomes more accessible and cost-effective, we have faced a growing demand from researchers who wish to use the Genome Browser tools to browse and annotate genome sequences for which we do not host a database. In response to this need, we have extended the functionality of track data hubs to encompass entire assemblies that are not hosted natively on the Genome Browser. These ‘assembly data hubs’ enable researchers to import both the underlying reference sequence as well as data tracks annotating that sequence into the Genome Browser for display and analysis. The genome sequence is stored in the UCSC .2bit format and made available on the user’s remote web server, along with optional annotation data files stored in the same compressed binary formats supported by track data hubs. Track and assembly data hubs can be shared with others by providing the URL of the hub.txt file needed to load the hub. Hubs of general interest to the research community can be registered at UCSC for sharing on the Genome Browser website. We offer a growing collection of publicly shared track and assembly data hubs on the ‘Public Hubs’ tab on the Genome Browser Track Data Hubs web page (http://genome.ucsc.edu/cgi-bin/hgHubConnect), including data sets from the ENCODE AWG, the Roadmap Epigenomics Project (22) and the Blueprint Epigenome Project (30). For more information about creating and using assembly data hubs, refer to http://genomewiki.ucsc.edu/index.php/Assembly_Hubs and http://genome.ucsc.edu/goldenPath/help/hgTrackHubHelp.html.

### Variant annotation integrator

To assist researchers in annotating and prioritizing thousands of variant calls from sequencing projects, we have developed a new software tool, the Variant Annotation Integrator (VAI). Given a set of variants uploaded as a custom track in either Personal Genome SNP (pgSnp) or VCF format, the VAI returns the predicted functional effect (e.g., synonymous, missense, frameshift, intronic) for each variant. The VAI can also provide several other types of relevant information, such as the dbSNP identifier if the variant is found in dbSNP, protein damage scores for missense variants from the Database of Non-synonymous Functional Predictions (dbNSFP) (31) and conservation scores computed from multiple-species alignments. Filters are available to focus results on the variants of greatest interest. The VAI can be accessed from the Genome Browser ‘Tools’ menu or through the VAI button on the ‘Manage Custom Tracks’ page that displays after a custom track is loaded into the Browser. For more information about the VAI, see http://genome.ucsc.edu/cgi-bin/hgVai.

### Gene haplotype alleles

**Figure 1.**
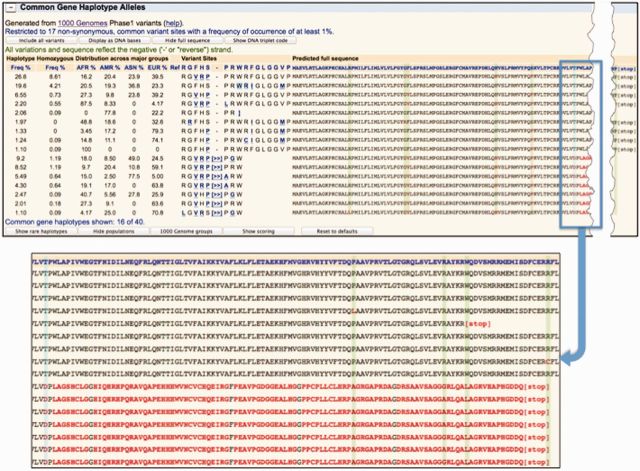
The haplotype alleles display for the ABO gene, which encodes proteins related to the ABO blood group system. A large portion of the ‘Predicted full sequence’ section is truncated in the upper image for display purposes, and is shown in greater detail in the lower image. The leftmost columns of the top image indicate the frequency of each allele haplotype within the 1000 Genomes sample and the occurrence of homozygosity for each allele. In this instance the haplotype alleles display has been expanded to show the distribution of the haplotypes across the major 1000 Genomes population groups. The ‘Variant Sites’ columns summarize the non-synonymous variant sites that occur in at least 1% of the subject chromosomes, with the value from the reference genome (in this case GRCh37/hg19) indicated at the top of each variant column. In all but one case, the ‘O’ phenotype results from a common insertion (indicated by ‘-’ in the reference) causing a frameshift (indicated by ‘[≫]’) that results in a downstream premature stop codon, thus truncating the protein. Note that although certain haplotyes are more frequently found within one population, the insertion that gives rise to the majority of ‘O’ phenotypes is found across all populations, which may indicate that the insertion predates the most recent migration out of Africa. On the other hand, the haplotype in which the SNP variant introduces a stop codon at the variant site may have arisen in the Americas. The zoomed-in view of the ‘Predicted full sequence’ section in the bottom image shows the reference sequence (top row) and sequences incorporating the common non-synonymous variants. The residues corresponding to the variant sites are highlighted by green vertical bars, the site corresponding to the frameshift-causing insertion is highlighted by a blue bar and changes to the reference amino acid sequence are shown in red.

We have extended the protein-coding genes detail pages in the UCSC Genes track on the GRCh37/hg19 human assembly to include a section that displays and compares ‘gene haplotype alleles’ generated from phased chromosomal data from Phase 1 of the 1000 Genomes Project (19) (Figure 1). Each haplotype allele is a distinct set of variants found on at least one of the 1000 Genomes subject chromosomes. By default the common non-synonymous variants (those of at least 1% frequency) are displayed, although rare haplotypes are optionally available. The Browser shows the frequency of each haplotype in the 1000 Genomes populations and indicates the frequency with which it occurs homozygously. Unexpected frequencies of occurrence may be used to identify alleles that merit further study. Predicted protein sequence for common haplotypes can also be displayed, allowing differences among alleles to be used to identify differences at the amino acid level. To access the gene haplotype alleles information, go to the details page for any protein-coding gene in the UCSC Genes track (GRCh37/hg19 assembly) and click the ‘Gene Alleles’ link in the ‘Page Index’ matrix. For more information, see http://genome.ucsc.edu/goldenPath/help/haplotypes.html.

### Updates to Browser display and navigation

During the past year, we have made several improvements to the Genome Browser web interface, many in response to requests from our users. We have updated the navigation menus for much of the website and simplified the background on the Genome Browser tracks page. The Browser now offers chromosome ideograms for genome assemblies that do not have a microscopically derived cytology. The drag-reorder feature in the Browser image now supports the vertical dragging of subtracks to any location in the image. We have also made display improvements to overlay wiggle tracks, and have improved the display speed of bigDataUrl custom tracks.

### User training and mirror support

We continually update and expand our documentation and training materials, which offer extensive information on using the Genome Browser tools to explore UCSC-hosted data sets as well as custom sequence and annotation data hosted at user sites. We are broadening our onsite training program to include several additional geographical regions. To better support our Genome Browser mirror sites and source code users, we have rearchitected the software makefile system for our utilities and command-line tools to allow the compilation of specific tools independent of a full Browser installation. We have also adopted UDR (https://github.com/LabAdvComp/UDR), a new package that integrates rsync with the high-performance network protocol UDT, allowing quicker transfers of our large data sets to remote mirror sites.

## FUTURE PLANS

During the upcoming year we will continue to add new and updated genome assemblies for vertebrate organisms as they become available in NCBI’s GenBank repository. We plan to release a preliminary Browser with a minimal annotation set on the new GRCh38/hg38 human assembly in late 2013 or early 2014. New annotation data display types and features will be added as required by new data sets. We plan to extend track hubs to support new file formats, such as the HAL hierarchical multiple alignment format (32), and to allow searching for tracks within a hub. The VAI will be expanded to include more input/upload options, output formats and annotation options.

## CONTACTING US

To stay on top of the latest Genome Browser announcements, genome assembly releases, new software features, updates and training seminars, subscribe to the genome-announce@soe.ucsc.edu mailing list or follow @GenomeBrowser on Twitter. We have two public, moderated mailing lists for interactive user support: genome@soe.ucsc.edu for general questions about the Genome Browser and genome-mirror@soe.ucsc.edu for questions specific to the setup and maintenance of Genome Browser mirrors. Messages sent to these lists are archived on public, searchable Google Groups forums. You may also reach us privately at genome-www@soe.ucsc.edu, the preferred address for inquiring about mirror site licenses, reporting server errors or contacting us about confidential issues. You will find complete contact information, links to the browser’s Google Groups forums and access to our user suggestion box at http://genome.ucsc.edu/contacts.html.

## FUNDING

This work was supported by the National Human Genome Research Institute [5U4 HG002371 to G.P.B., H.C., J.C., T.R.D., P.A.F., L.G., S.H., A.S.H., M.H., D.K., W.J.K., R.M.K., K.L., B.T.L., C.H.L, B.J.R., B.R., M.L.S. and A.S.Z.; 1U41HG006992 subcontract 60141508-106846-A to D.K., W.J.K., K.L., M.S.C., T.R.D., B.J.R., K.R.R., C.A.S. and A.S.Z]; National Institute of Dental and Craniofacial Research [5U01DE020057 subcontract 1000736806 to G.P.B. and R.M.K.]; National Cancer Institute [1U41HG007234 subcontract 2186-03 to M.D. and R.H.; 5U24CA143858 to M.C.]. European Molecular Biology Organization Long-Term Fellowship (in part) [ALTF 292-2011 to M.H.]; Howard Hughes Medical Institute fellow (to D.H.). Funding for open access charge: National Human Genome Research Institute.

*Conflict of interest statement*. G.P.B., H.C., M.D., T.R.D., P.A.F., L.G., D.H., R.A.H., S.H., A.S.H., D.K., W.J.K., R.M.K., K.L., C.H.L., B.J.R., B.R., K.R.R., C.A.S. and A.S.Z. receive royalties from the sale of UCSC Genome Browser source code licenses to commercial entities. W.J.K. works for Kent Informatics.
